# Supplementary figures and images for: Investigating Causal Associations of Circulating Micronutrients Concentrations with the Risk of Lung Cancer: A Mendelian Randomization Study
Source: Nutrients. 2022 Oct 31;14(21):4569. doi: 10.3390/nu14214569 (PMC9655558; doi:10.3390/nu14214569)

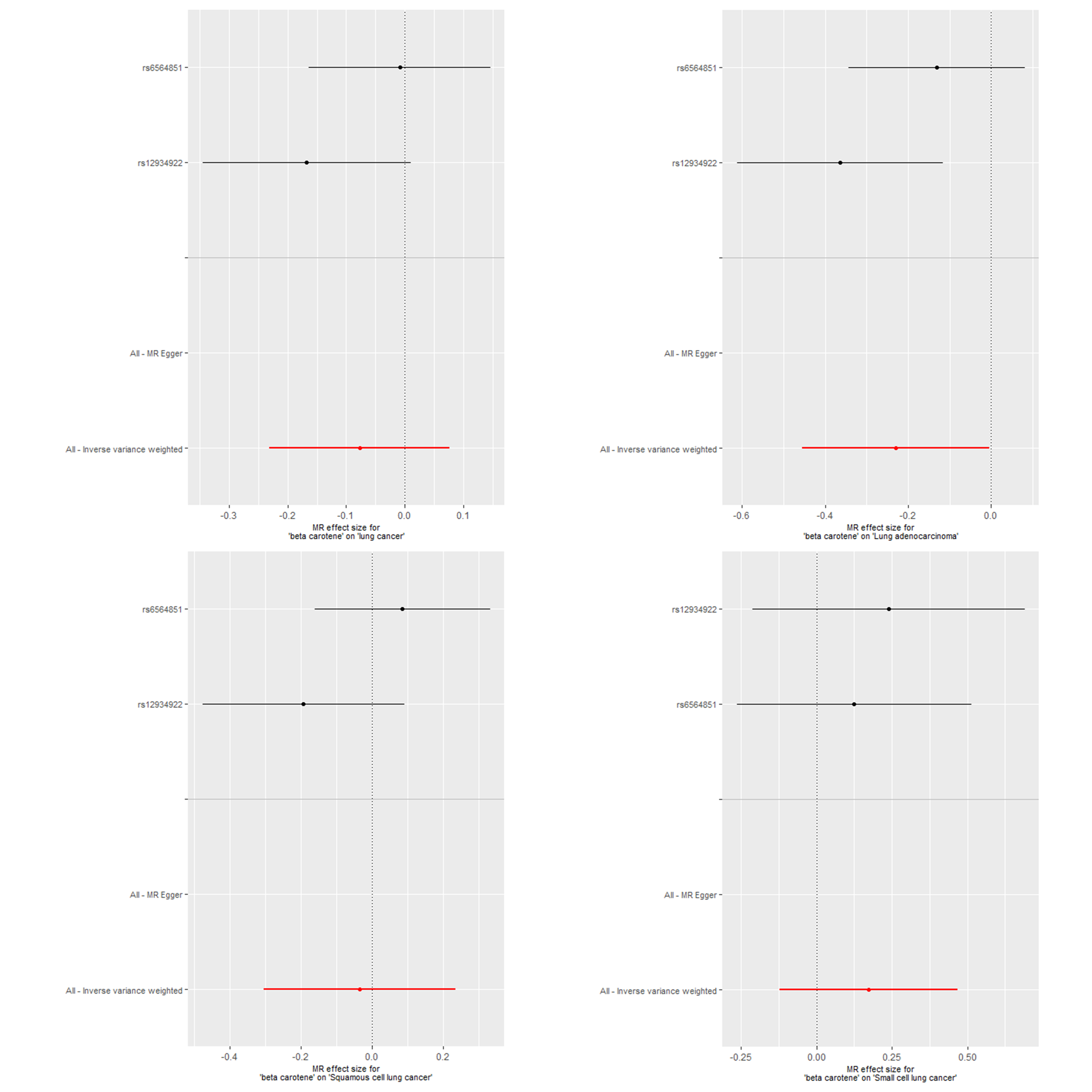

Supplement: Supplementary file 1 [file nutrients-14-04569-s001.zip › Supplementary figures/Figure S1.tif]

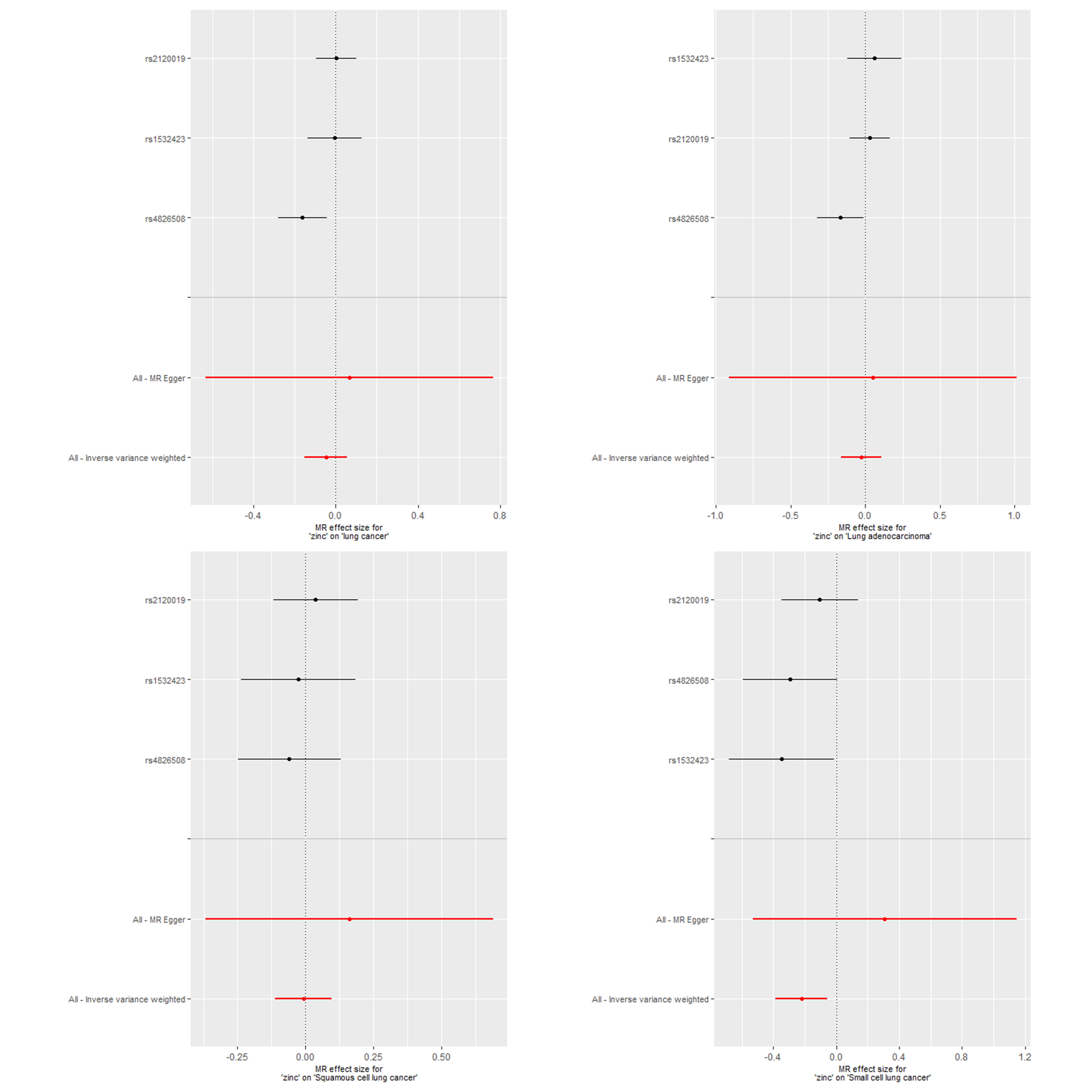

Supplement: Supplementary file 1 [file nutrients-14-04569-s001.zip › Supplementary figures/Figure S10.tif]

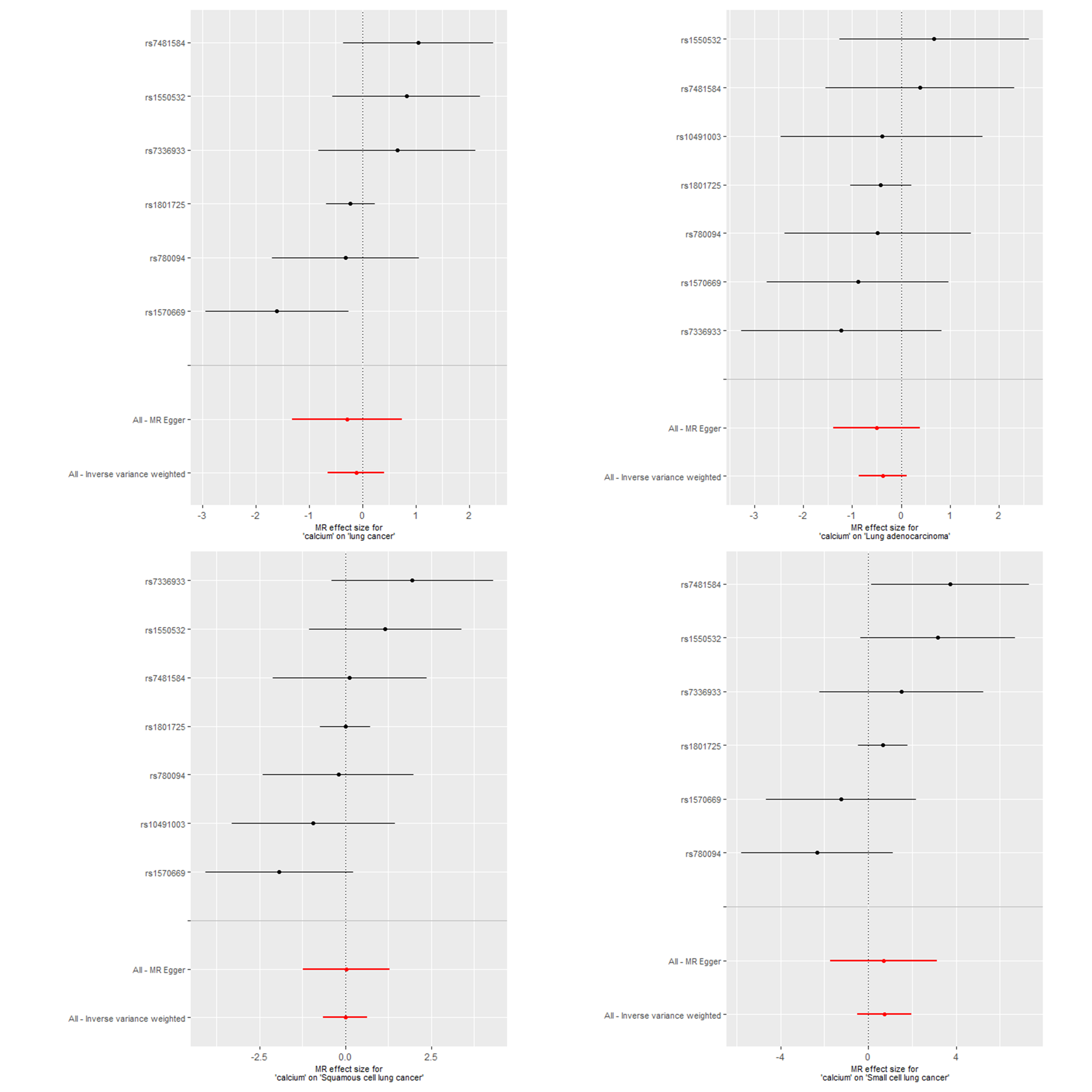

Supplement: Supplementary file 1 [file nutrients-14-04569-s001.zip › Supplementary figures/Figure S2.tif]

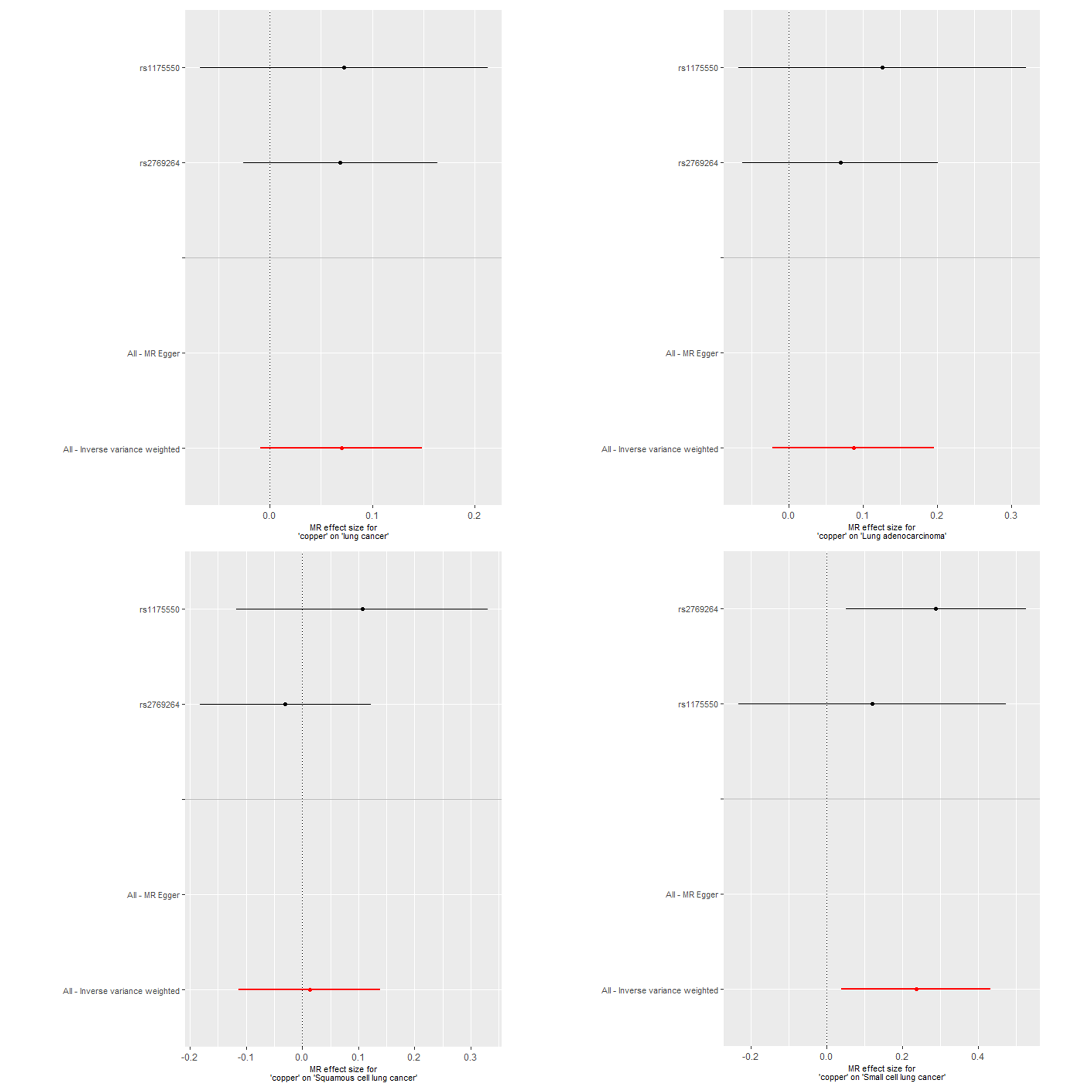

Supplement: Supplementary file 1 [file nutrients-14-04569-s001.zip › Supplementary figures/Figure S3.tif]

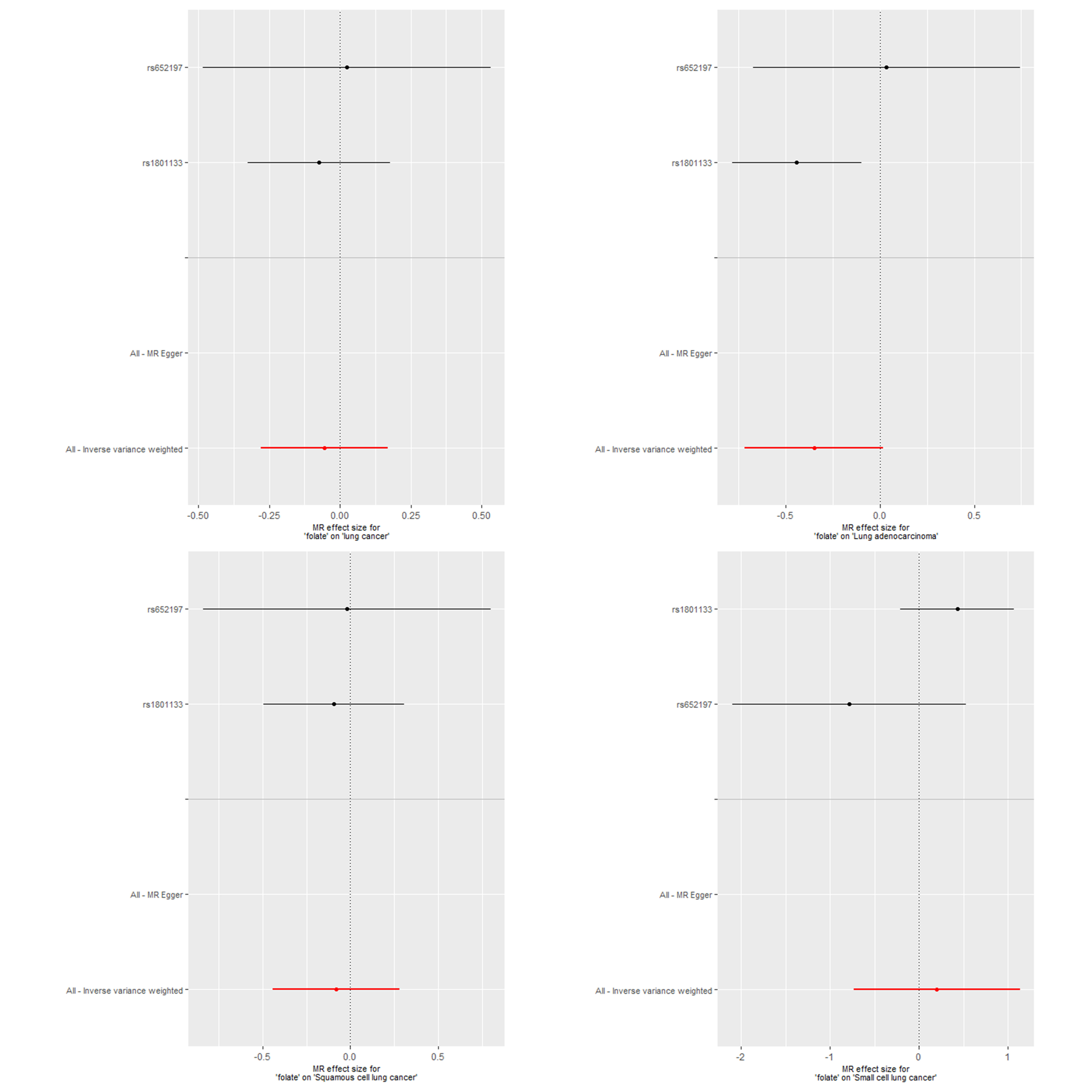

Supplement: Supplementary file 1 [file nutrients-14-04569-s001.zip › Supplementary figures/Figure S4.tif]

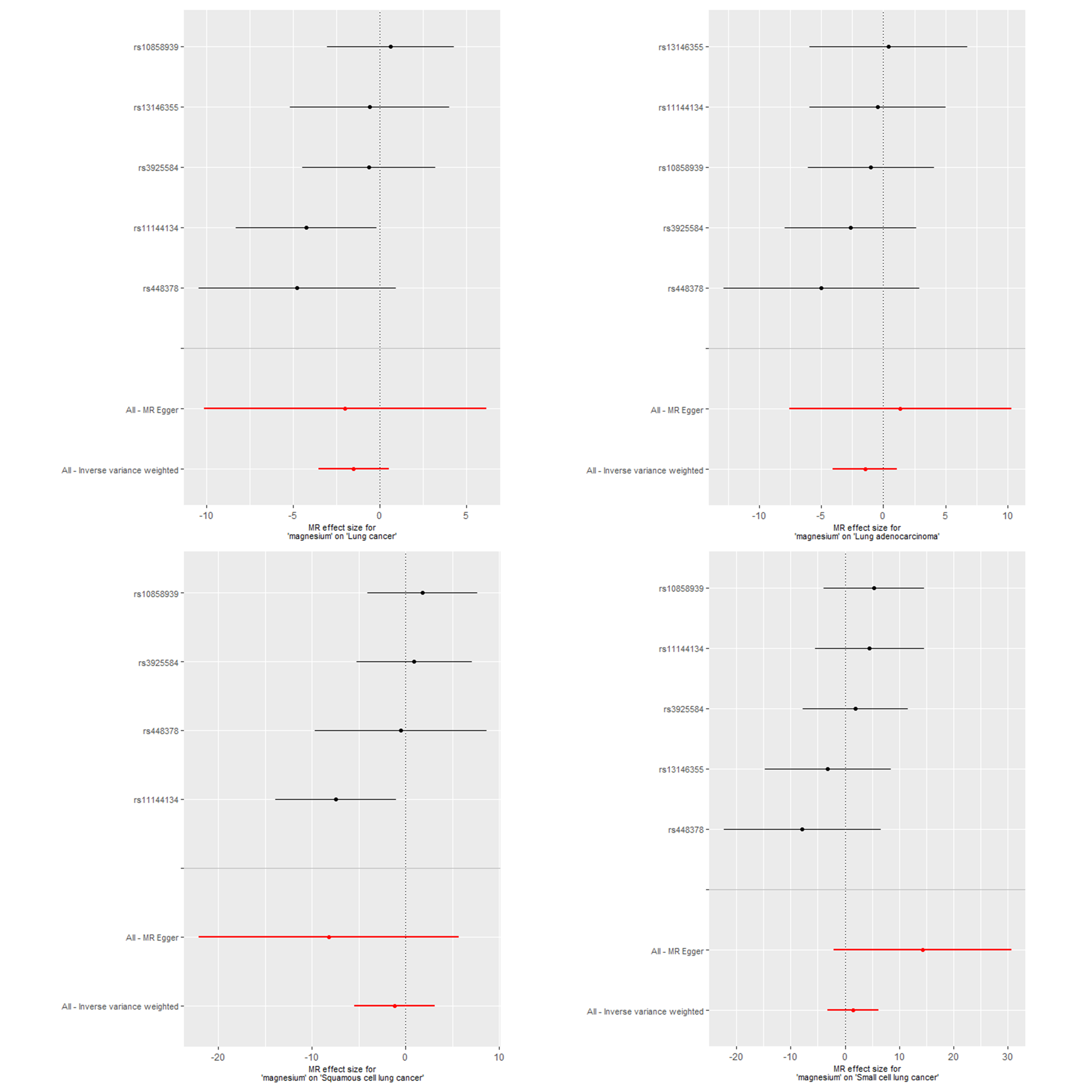

Supplement: Supplementary file 1 [file nutrients-14-04569-s001.zip › Supplementary figures/Figure S5.tif]

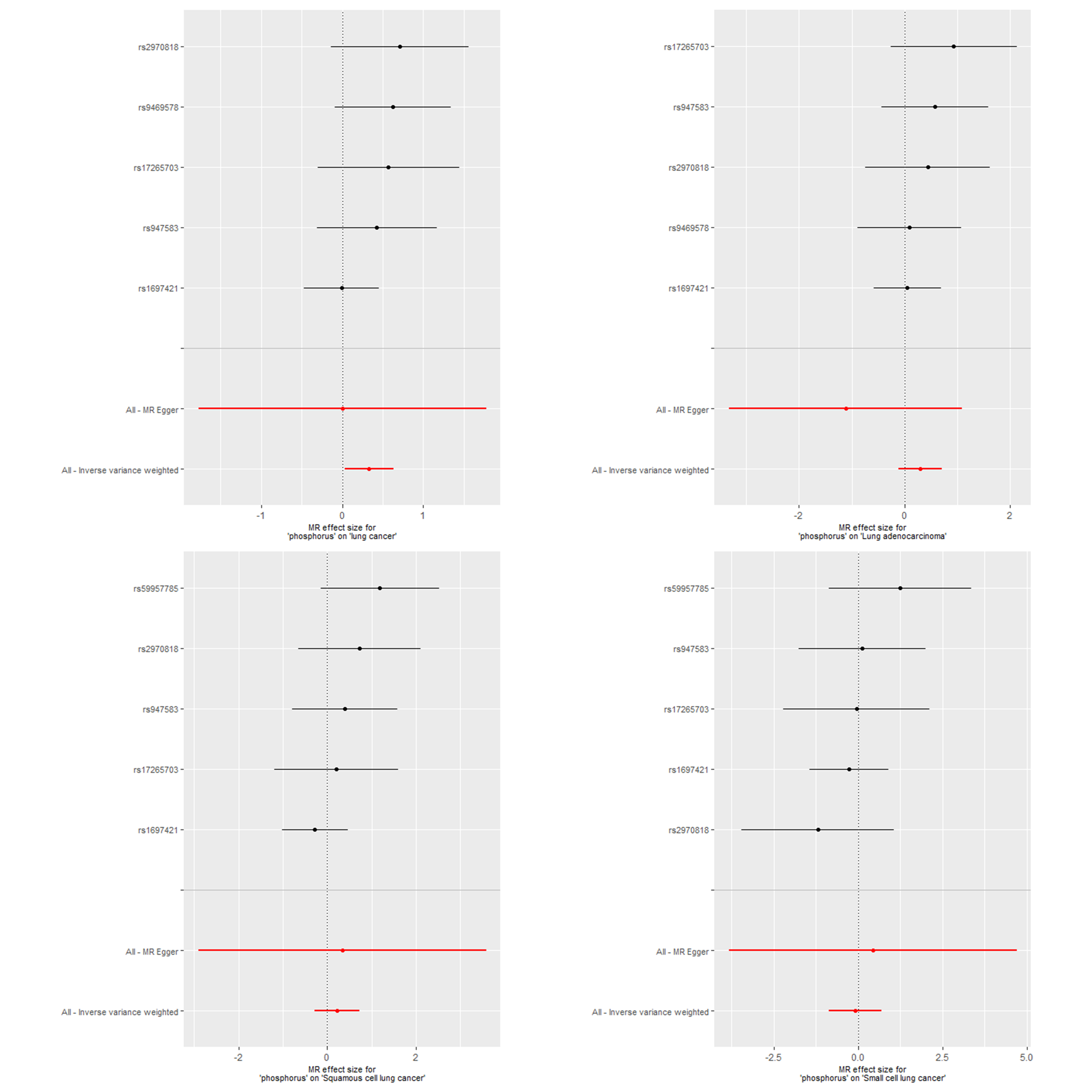

Supplement: Supplementary file 1 [file nutrients-14-04569-s001.zip › Supplementary figures/Figure S6.tif]

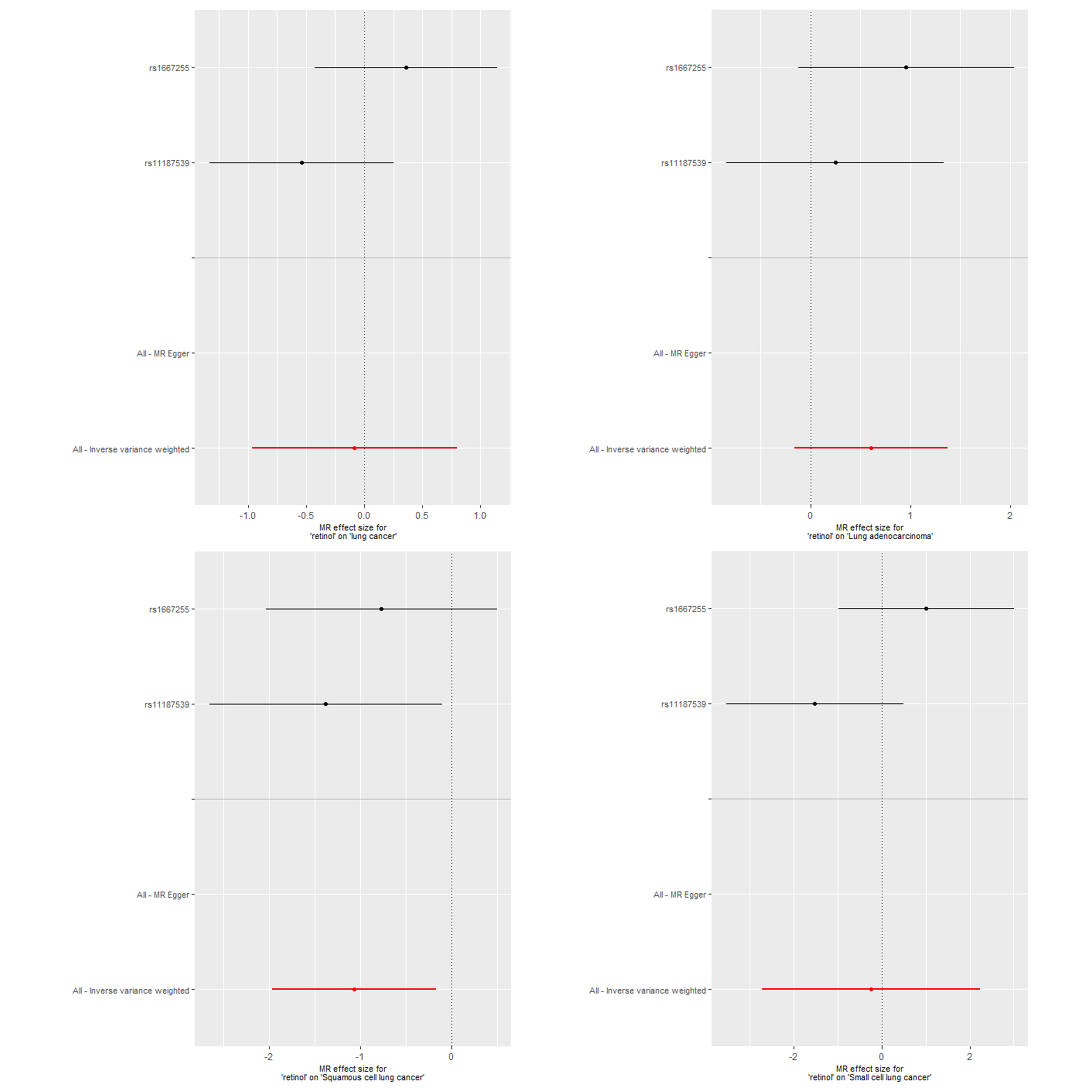

Supplement: Supplementary file 1 [file nutrients-14-04569-s001.zip › Supplementary figures/Figure S7.tif]

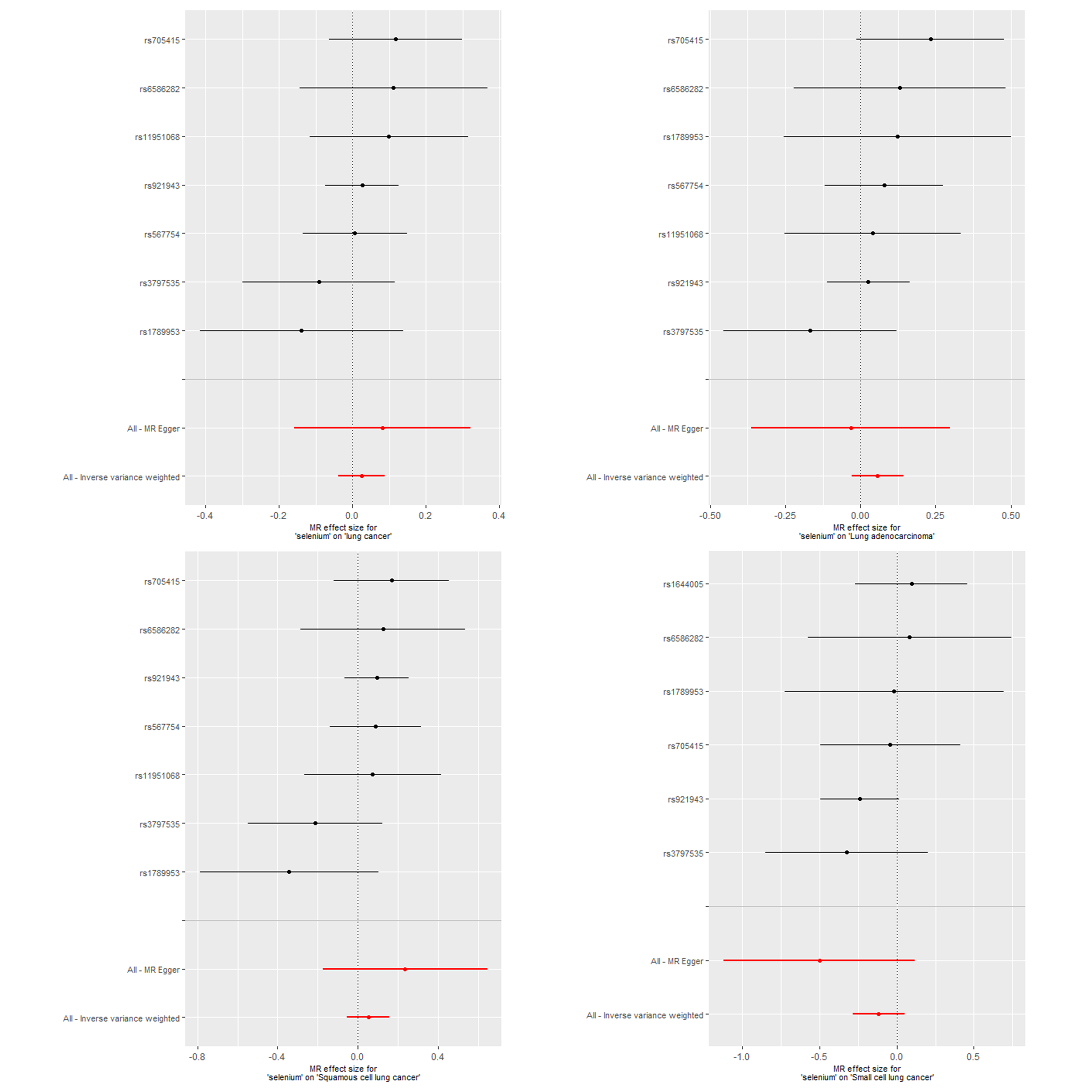

Supplement: Supplementary file 1 [file nutrients-14-04569-s001.zip › Supplementary figures/Figure S8.tif]

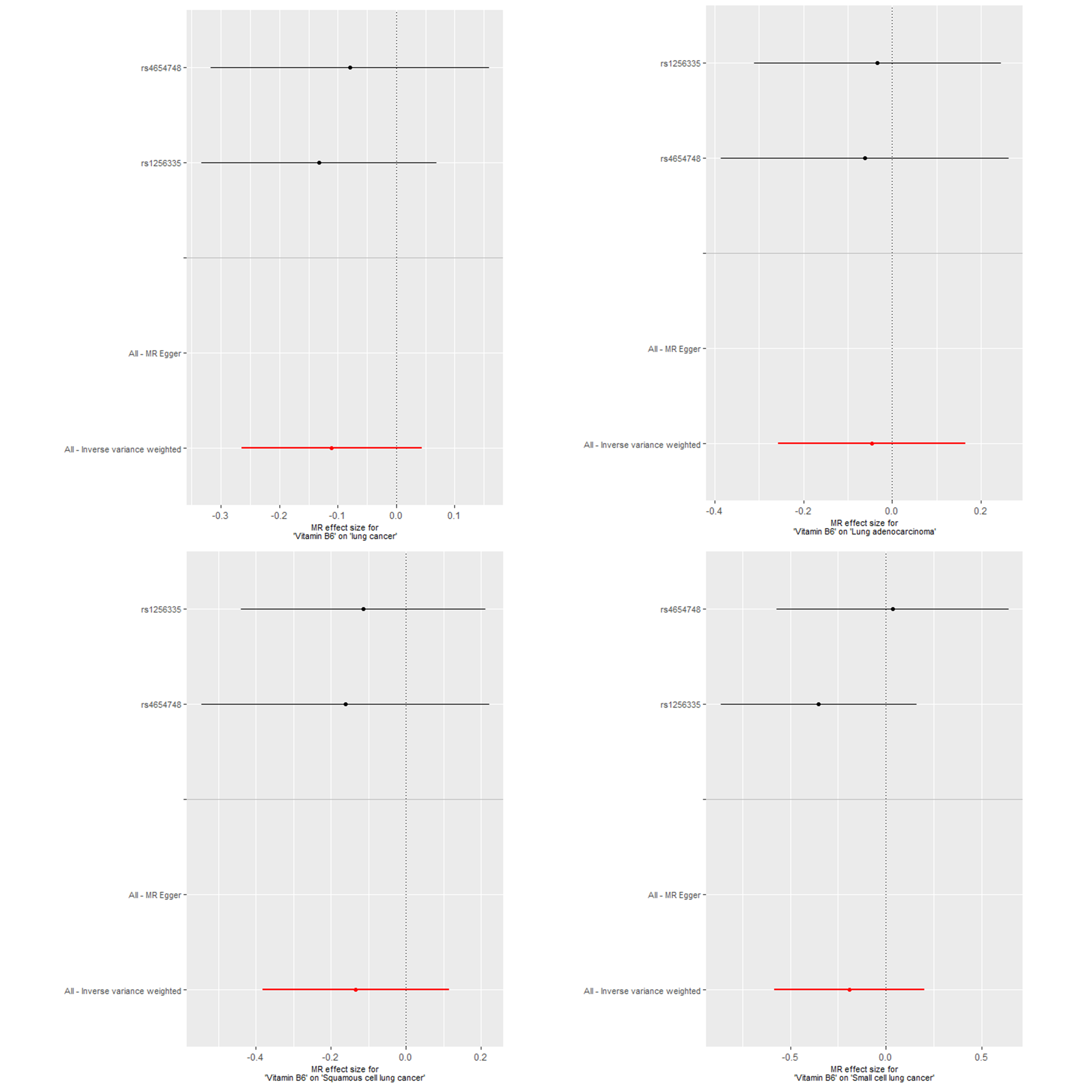

Supplement: Supplementary file 1 [file nutrients-14-04569-s001.zip › Supplementary figures/Figure S9.tif]
